# Nutritional and Functional Enhancement of Chinese Steamed Bread Through Incorporation of *Acheta domesticus* and *Antheraea pernyi* Pupae Powders

**DOI:** 10.3390/foods14223956

**Published:** 2025-11-19

**Authors:** Yu Liu, Yangran Lu, Poompatchara Nakkote, Hua Li, Ruixin Liu, Sirithon Siriamornpun

**Affiliations:** 1 Department of Cuisine and Nutrition, Yangzhou University, Yangzhou 225127, China; mx120231288@stu.yzu.edu.cn (Y.L.); mx120251324@stu.yzu.edu.cn (Y.L.); lihua216@yzu.edu.cn (H.L.); 2 Faculty of Economics, Khonkaen University, Mueang 40002, Khon Kaen, Thailand; poomna@kku.ac.th; 3 Key Laboratory of Chinese Cuisine Intangible Cultural Heritage Technology Inheritance, Ministry of Culture and Tourism, Yangzhou 225127, China; 4 Research Unit of Thai Food Innovation, Department of Food Technology and Nutrition, Mahasarakham University, Kantarawichai 44150, Maha Sarakham, Thailand

**Keywords:** Chinese steamed bread, edible insect, biological activity, starch digestibility, volatile flavor compound, texture property, sensor response pattern

## Abstract

The study intended to assess the impacts of partially replacing wheat flour with *Acheta domesticus* powder (AD) and *Antheraea pernyi* pupae powder (AP) at 5%, 10%, and 15% levels on the proximate composition, color properties, texture profile, antioxidant capacity, starch digestibility, and flavor characteristics of Chinese steamed bread (CSB). The addition of AP and AD notably increased the levels of protein, fat, and ash and also led to an elevated chewiness and hardness. Most importantly, compared to the control, AP- and AD-fortified CSB exhibited a significantly low estimated glycemic index (eGI) and high total phenolic and flavonoid contents, resulting in improved DPPH and ABTS radical scavenging activities. Furthermore, E-nose, E-tongue, and GC-MS analysis revealed that incorporation of AP and AD strengthened umami and saltiness and enriched the flavor profile of CSB. Our findings indicate that AD- and AP-fortified CSB is a promising functional food with a lower eGI, as well as improved nutritional value and antioxidant potential, offering a sustainable strategy for staple food innovation while also providing guidance for consumers to select wheat flour fortified with different types and levels of insect powder to prepare CSB based on their preferences.

## 1. Introduction

Chinese steamed bread (CSB) is a renowned traditional staple food distinguished by a soft texture and a mellow taste [1]. It is produced by steaming a leavened dough consisting of wheat flour and water [2]. Although refined wheat flour products are preferred for their palatability, they are not regarded as part of a healthy diet owing to their high glycemic index and lack of essential amino acids [3]. To enhance the diversity, nutritional value, and consumer appeal of CSB, researchers have developed novel CSB formulations through the incorporation of functional ingredients, such as edible plants (e.g., legumes [4], red beetroot [5], dock [6], yams [7], and potato [8]) and dietary fibers (e.g., inulin [9]).

In response to global demands for sustainable protein sources, insect-derived ingredients have emerged as promising alternatives. Compared with conventional livestock farming, insect rearing demands substantially smaller quantities of land, water, and feed and is associated with far lower greenhouse gas emissions, which aligns with global sustainability goals [10]. One edible insect used as a food ingredient is *Antheraea pernyi* pupae. In China, the consumption of *A. pernyi* pupae has been engraved into the history of certain regions, and it has been approved as a common food in China by the National Health Commission since 2004 [11]. They are used for the preparation of protein concentrates and oil, or for fortifying noodles after being dried and milled [12]. *A. pernyi* pupae can provide high-quality proteins, fat (abundant in α-linolenic acid, oleic acid, and palmitic acid), and minerals (selenium). Another edible insect, *Acheta domesticus*, contains approximately 60% protein (with an essential amino acid score ranging from 0.96–1.02), 20% fat (rich in linoleic acid, oleic acid, and palmitic acid), dietary fiber, vitamins, and minerals (phosphorus and potassium) [13,14]. *A. domesticus* powder has been used for the enrichment of biscuits, pasta, and bread [15]. The European Union’s approval of this species for human consumption has further accelerated functional food innovation. In this regulatory context, the European Food Safety Authority has defined maximum usage levels for its powder across various food categories, including 100% for non-chip snacks, 50% for meat imitates, 16% for meat balls, 15% for cereal bars, 10% for bread and rolls with special ingredients added, 8% for biscuits, and 5% for various soups [16]. Moreover, Thailand has established both good agricultural practices for cricket farming and food regulations to further standardize cricket production, processing, and consumption throughout the supply chain.

However, their integration into mainstream diets is often hindered by cultural prejudices and sensory aversions [17]. Insects may exhibit a distinct aromatic character, defined by intense crustacean, cooked legume, and earthy notes [18], which pose challenges to their development and promotion in functional products. Most studies have concentrated on their chemical properties and nutritional profile, with limited attention paid to their flavor profiles. Notably, studying these flavor profiles is crucial for improving product quality and aligning the taste with target consumers’ preferences.

In this study, *A. domesticus* and *A. pernyi* pupae powders were incorporated into CSB at different concentrations to investigate their impacts on proximate composition, color, texture, and antioxidant properties. Additionally, starch digestibility, Fourier Transform Infrared Spectroscopy (FTIR) spectral characteristics, and flavor profile were also evaluated. This investigation was anticipated to offer valuable insights for developing sustainable, protein-dense food products that reconcile dietary tradition with modern nutritional needs.

## 2. Materials and Methods

### 2.1. Materials and Chemicals

*A. domesticus* powder (AD) was kindly provided by the Newgensinnofoods Co., Ltd. (Kantarawichai, Thailand), with a particle size of passing through a 250 µm screen. *A. pernyi* pupae were supplied by a sericulture farm (Benxi, China). All-purpose wheat flour was obtained from local supermarket. High-activity dry yeast was purchased from Angel Yeast Co., Ltd. (Yichang, China). DPPH, ABTS, gallic acid, and rutin were supplied by Yuanye Biotech (Shanghai, China). Amyloglucosidase and α-amylase were acquired from Aladdin Biotech (Shanghai, China). All other chemicals were of analytical grade and purchased from Sinopharm Chemical Reagent Co., Ltd. (Shanghai, China)

### 2.2. Preparation of A. pernyi Pupae Powder

*A. pernyi* pupae were lyophilized (LYOQUEST-55, Azibil Telstar, Shanghai, China), powdered, and sieved through a 180 µm screen. The *A. pernyi* pupae powder (AP) was stored at −20 °C until used.

### 2.3. Preparation of CSB

CSB samples were prepared following the protocol established by Yang et al. [19], with slight adjustments. The basic ingredients for CSB preparation were as follows: 100 g wheat flour, 0.8 g dry yeast, and 45 mL water. Dry yeast was dissolved in 37 °C water, and the resulting mixture was added to wheat flour and kneaded manually for 10 min. Followed by fermentation (37 °C, 85% RH, 30 min), the dough underwent steaming for 15 min, and finally cooled at room temperature for 1 h. For CSB containing insect powder, wheat flour was partially replaced by either AP or AD at 5%, 10%, and 15% levels. Freshly prepared CSB was used for color characteristics, texture properties, E-tongue, E-nose, and GC-MS analysis. For other measurements, the CSB was lyophilized and kept at −20 °C.

### 2.4. Proximate Composition Analysis

Moisture, protein, fat, and ash of CSB were measured using Chinese national standards GB 5009.3-2016 [20], GB 5009.5-2016 [21], GB 5009.6-2016 [22], and GB 5009.4-2016 [23], respectively. The total level of carbohydrates was calculated by the difference method.

### 2.5. Determination of Color

The crumb color of CSB was quantified by measuring L*, a*, and b* values with a colorimeter (CR-400, Konica Minolta, Tokyo, Japan) [24], following calibration with white and black standards prior to each measurement.

### 2.6. Determination of Texture Property

The texture profile of CSB was assessed as described by Shang et al. [25]. The sample slices (10 mm thickness) were centrally positioned on the texture analyzer fitted with a P/36R probe (TA-XT Plus, Stable Micro Systems, Godalming, UK). The instrument was configured with a 50% compression ratio, a universal speed of 1 mm/s for all phases, a 5 g trigger force, and a 5 s recovery interval between compressions.

### 2.7. Antioxidant Activity Analysis

#### 2.7.1. Preparation of Extracts

For polyphenol extraction, 0.5 g of sample was mixed with 25 mL of 70% methanol and extracted with orbital shaking (40 °C, 160 rpm, 2 h). Following centrifugation (3000 rpm, 20 min), the resulting supernatant was preserved at 4 °C for further use [26].

#### 2.7.2. Total Phenolic Content

The total phenolic content (TPC) of the samples was assayed following the procedure of Zhu et al. [2], with minor adjustments. The mixture of the extract (1 mL) and 10% Folin–Ciocalteu solution (4.5 mL) was incubated for 10 min. After the addition of 7.5% Na_2_CO_3_ (5 mL), the solution was stored in the dark for 2 h. Then, absorbance measurement was performed at 765 nm using a microplate reader (Ensight^TM^, PerkinElmer, Waltham, MA, USA). Finally, the results were reported in mg of gallic acid equivalents per 100 g of dry weight CSB (mg GAE/100 g).

#### 2.7.3. Total Flavonoid Content

The total flavonoid content (TFC) of the samples was determined with the aluminum chloride colorimetric method [27]. Briefly, the extract (1 mL) was reacted with 5% NaNO_2_ (0.3 mL) for 6 min. After adding 10% AlCl_3_ (0.3 mL), the mixture was maintained for 6 min. Then, 1 mol/L NaOH (4 mL) was added, and absorbance measurement was performed at 510 nm. The results were reported in mg of rutin equivalents per 100 g of dry weight of CSB (mg RE/100 g).

#### 2.7.4. DPPH Radical Scavenging Activity

The DPPH value was measured via the method of Khuku et al. [28]. In brief, the mixture of the extract (0.5 mL) and DPPH solution (3.5 mL) was incubated at room temperature for 30 min in the dark. Then, the absorbance at 515 nm was obtained using a spectrophotometer (V-1100D, Mapada, Shanghai, China). The scavenging activity was determined as a percentage, employing the following equation:

(1) $$DPPH(\%)=\frac{A_{1}-A_{2}}{A_{1}}\times 100$$

where A_1_ and A_2_ correspond to the absorbance of the DPPH solution mixed with ethanol and with the sample extract, respectively.

#### 2.7.5. ABTS Radical Scavenging Activity

The ABTS value was measured via the method of Xu et al. [26], with minor adjustments. The absorbance of the working solution (prepared from ABTS and potassium persulfate) was adjusted to 0.7 ± 0.02 at 734 nm. Subsequently, 0.1 mL of the extract was combined with 3.9 mL of the working solution, and the absorbance at 734 nm was obtained after a 10 min reaction time.

(2) $$ABTS(\%)=\frac{A_{1}-A_{2}}{A_{1}}\times 100$$

where A_1_ and A_2_ correspond to the absorbance of the ABTS solution mixed with distilled water and with the sample extract, respectively.

### 2.8. In Vitro Starch Digestibility Analysis

The estimated glycemic index (eGI) and starch composition were investigated via the method of Li et al. [29]. The CSB sample (200 mg) was dispersed in 15 mL of sodium acetate buffer (0.2 mol/L, pH 5.2) and boiled at 100 °C for 15 min. The resulting mixture was then digested with amyloglucosidase and α-amylase at 37 °C and 160 rpm for 3 h in a water bath (SHZ-82A, Shengwei Instruments, Changzhou, China). During the incubation, the glucose level in the digesta was quantified via the DNS method at 0, 20, 30, 60, 90, 120, 150, and 180 min. The hydrolysis index (HI) for each CSB sample was determined by dividing its AUC by that of fresh white bread and was expressed as a percentage. The eGI was then derived based on the below equation:

(3) eGI = 8.198 + 0.862 × HI

### 2.9. FTIR Analysis

The FTIR spectra of the CSB samples were obtained using the method of Yan et al. [30], with slight adjustments. The freeze-dried CSB powder was thoroughly combined with KBr at a 1:10 (*w*/*w*) ratio and ground to a homogeneous mixture. The spectra were collected from 4000 to 400 cm^−1^ with a resolution of 4 cm^−1^.

### 2.10. E-Nose Analysis

The volatile compounds in the CSB samples were characterized by an E-nose (PEN3, Airsense Analytics Inc., Schwerin, Germany), based on Chi’s procedure with minor modifications [31]. The corresponding sensitive substances of 10 metal sensors are described in Table 1. Prior to analysis, the CSB sample was heated in a sealed 40 mL headspace vial at 60 °C for 30 min to establish equilibrium. The instrument parameters were set as follows: a common flow rate of 400 mL/min for both the chamber and injection, with time settings of 1 s (sample interval), 60 s (flush), 10 s (zeroing), 5 s (pre-sampling), and 60 s (measurement).

### 2.11. GC-MS Analysis

The profile of volatile organic compounds (VOCs) was characterized by headspace solid phase micro-extraction technique (HS-SPME) coupled with GC-MS, according to the procedure outlined by Xi et al. [32]. HS-SPME extraction was conducted via a 75 μm CAR/PDMS fiber. Prior to extraction, the fiber was aged at 250 °C for 15 min, and then exposed to the sample headspace at 60 °C for 30 min. Following a 5 min thermal desorption of the fiber at 250 °C, the analytes were subsequently introduced into the GC-MS system for separation and identification. Chromatographic separation was achieved using a DB-5 MS column (30 m × 0.25 mm × 0.25 μm) with helium as the carrier gas (1.0 mL/min) and an inlet temperature of 250 °C in splitless mode. The oven temperature was held at 40 °C for 2 min, then increased to 100 °C at 5 °C/min, further raised to 250 °C at 5 °C/min, and finally ramped to 280 °C at 10 °C/min and held for 5 min. The MS was operated with an electron ionization source at 250 °C and 70 eV electron energy. Qualitative and quantitative analyses were conducted in selected ion monitoring mode, with mass scans acquired covering *m*/*z* 35–450. Volatile compounds were characterized by comparing their mass spectra with those in the NIST library; a match quality above 85% was considered confirmed. The relative content of each compound was expressed as its percentage contribution to the total peak area.

### 2.12. E-Tongue Analysis

The taste profile analysis was carried out via an SA402B E-tongue (Intelligent Sensor Technology Co., Ltd., Atsugi, Japan) based on a modified protocol [33]. The taste sensing system comprised eight parameters: primary tastes (saltiness, umami, bitterness, and sourness), mouthfeel (richness and astringency), and aftertaste components (aftertaste astringency, aftertaste-A; aftertaste bitterness, aftertaste-B). To perform this measurement, 15 g of the CSB samples was homogenized with 150 mL of distilled water and then subjected to centrifugation (10,000 rpm, 25 min, 4 °C). Afterwards, 40 mL of the resulting supernatant was detected. Each sample was subjected to four measurement cycles, with the first cycle’s data discarded and the subsequent three retained for analysis.

### 2.13. Statistical Analysis

All analyses were conducted at least in triplicate. The results were presented as mean ± standard deviation. Statistical analysis was performed using one-way analysis of variance (ANOVA) followed by Duncan’s multiple range tests, with a *p*-value < 0.05 considered statistically significant.

## 3. Results and Discussion

### 3.1. Nutritional Composition

Table 2 compares the effects of incorporating two kinds of edible insect (AP and AD) at varying substitution levels on the proximate composition of CSB. The proximate composition of AP and AD was described in our earlier publications [12,34], as shown in Table S1. For both types of edible insect, the protein content of CSB significantly elevated with higher substitution levels in comparison to the control (*p* < 0.05). Specifically, the incorporation of 15% AP and 15% AD caused a considerable rise in protein content by 61.50% and 76.16%, respectively. The increase is likely due to the relatively lower protein content in wheat flour than that in both AP and AD [35]. This result agrees with the observations from a prior study, which revealed that the incorporation of 10% *Alphitobius diaperinus* powder in cookies resulted in a 26.91% increase in protein content [36]. Another study by Biró et al. [37], which observed a 55.17% rise in the protein content of oat biscuits incorporated with 15% *A. domesticus* powder, further supports our findings.

Moreover, substitution with 15% AP significantly increased the fat and ash contents by 4.86-fold and 2.68-fold, respectively, compared to the control. Similarly, 15% AD substitution led to increases 3.35-fold in fat and 4.13-fold in ash content. This increase in fat content was due to the higher fat content in insect powder than in wheat flour. Based on the fat content analysis, the utilization of defatted insect powder is recommended to develop healthier products, as advocated by Roncolini et al. [38]. The ash level corresponds to the mineral makeup of a food. These results suggest that fortification with insect powder effectively enhanced the mineral content of CSB compared to conventional wheat flour-based products. Kowalski et al. [39] also found that high inclusion levels (i.e., 15% and 30%) of insect powder could increase the fat and ash contents of nut bars. With increasing substitution of AP or AD, the carbohydrate content in CSB decreased, attributable to the lower carbohydrate content in insect powder compared to wheat flour.

### 3.2. Color

**Figure 1:**
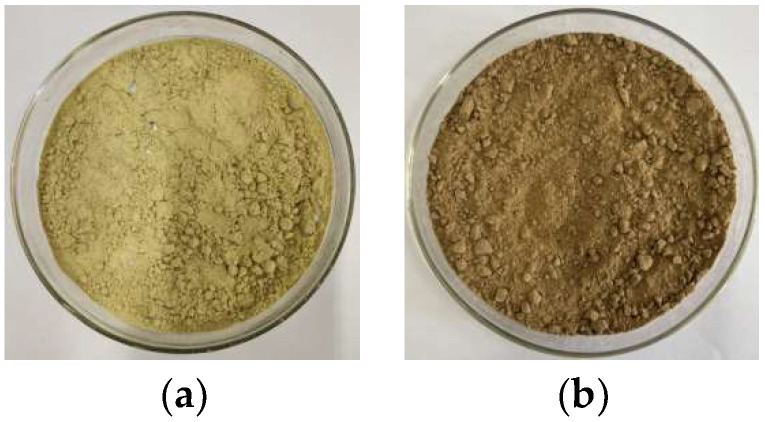
Appearance of (**a**) *Antheraea pernyi* pupae powder and (**b**) *Acheta domesticus* powder.

Table 3 presents the color parameters of freshly prepared CSB. Color plays a key role in determining consumer acceptance of foods, since visual assessment often serves as the primary criterion for product recognition [40]. Generally, the L* values of CSB fortified with AP and AD substantially reduced compared to the control. At the same inclusion levels, CSB fortified with AD exhibited lower L* values than those fortified with AP, which could be explained by the inherently darker color of AD compared to AP (see Figure 1). In contrast, the a* and b* value increased after incorporating insect powder into CSB, indicating a shift in color chromaticity toward red and yellow. However, no significant differences were observed in a* value among control, AP5, AD5, and AP10. Although AD supplementation caused a slight increase in b* values, no significant difference (*p* < 0.05) was found compared to the control. The phenolic compounds of AP or AD may undergo oxidation during the steaming process, resulting in color changes. Moreover, the high protein concentration in insect powder promotes Maillard reactions during steaming, leading to the formation of melanoidins and other browning pigments, which contribute to the increased yellowness of CSB [28,41]. These trends were in line with the findings of Wang et al. [42], who reported that increasing the content of rose powder resulted in darker-colored CSB, along with elevated a* and b* values. The change in color may enhance consumers’ preference by suggesting freshness.

### 3.3. Textural Property

The textural attributes of CSB are closely relevant to the mouthfeel [26], which is a critical factor affecting consumer acceptance. According to Table 4, the hardness and chewiness of CSB increased progressively with higher incorporation levels of insect powder. Conversely, a general decreasing trend was noted in springiness, cohesiveness, and resilience. The increase in protein content promoted stronger interactions between the incorporated insect protein and wheat gluten, resulting in the formation of a more dense and rigid structure that ultimately enhanced the hardness of the CSB [43]. Similarly, Bhatnagar et al. [44] observed that protein supplementation increased the hardness of buns, which could be ascribed to the reduced gluten content in fortified products relative to the control. With the level of AD at 15%, the hardness and chewiness of CSB increased markedly, resulting in a less desirable mouthfeel. Due to the high energy and time demands of oral processing prior to swallowing, excessive chewiness and hardness can be detrimental to the palatability of CSB [42]. However, changes in the texture of CSB induced by edible insect powder substitution do not necessarily reduce consumer acceptance. Consumer preferences for the texture of CSB exhibit regional variations [2], highlighting the potential of edible insects to improve CSB quality.

### 3.4. Antioxidant Activity

Indeed, numerous bioactive compounds such as carotenoids, tocopherols, lignans, flavonoids, phytosterols, and phenolic acids are naturally present in wheat [2]. The TPC and TFC of CSB fortified with AP or AD increased dose-dependently with insect powder inclusion. As displayed in Table 5, TPC ranged from 5.63 to 32.66 mg GAE/100 g, with the highest increase (5.80 fold) observed in CSB containing 15% AP. Similarly, TFC increased significantly in fortified samples, ranging from 3.85 to 12.59 mg RE/100 g, compared to 0.45 mg RE/100 g in the control CSB. These results are in line with those found by Ogidi et al. [45], who demonstrated that the fortification of 10% *Macroterms nigeriensis* into wheat cookies significantly improved both TPC and TFC.

The AP15 group showed the highest improvement in antioxidant activity, with DPPH and ABTS values reaching 4.9 and 2.8 times higher than the control, respectively. The AD15 group also exhibited strong enhancement, achieving 3.2-fold and 2.2-fold increases in DPPH and ABTS values. The most pronounced improvements were observed at 10–15% fortification, consistent with the elevated polyphenol content—a phenomenon potentially attributable to insects’ abundance in bioactive constituents like chitins, tocopherols and ferulic acid [12]. Additionally, the formation of Maillard reaction products during the CSB manufacturing process, which are known for their antioxidant activity [46], further contributed to the enhanced antioxidant capacity. The radical scavenging activities significantly elevated in the AP- and AD-fortified CSB, compared to the control, which is in line with the findings of our previous study on the influence of edible insect incorporation in rice noodles [47]. Nissen et al. [46] obtained similar trends after adding cricket flour in gluten free sourdough bread. These results suggested that insect powder fortification not only improved the nutritional value but also enhanced the antioxidant profile of CSB.

### 3.5. In Vitro Starch Digestibility

Table 6 displays the eGI and starch composition of CSB fortified with AP or AD. The control exhibited the highest rapidly digestible starch (RDS) content (58.87%), which significantly decreased with higher levels of AP or AD incorporation (*p* < 0.05). In contrast, slowly digestible starch (SDS) and resistant starch (RS) contents increased with the addition of AP or AD. Compared to the control, the AP15 group exhibited increases of 12.61% in SDS and 103.32% in RS, while the AD15 group showed increases of 13.48% and 122.92%, respectively. The incorporation of insect powder considerably lowered the eGI of CSB in a dose-dependent fashion. In comparison with the control, the incorporation of 15% AP and 15% AD resulted in a reduction in eGI by 20.55% and 23.71%, respectively. These results indicated that the addition of insect powder effectively inhibited starch digestion. This phenomenon could be explained by the elevated levels of protein, lipid, and polyphenols in insect-fortified CSB. Proteins and polyphenols retard starch digestibility through two primary mechanisms: firstly, by suppressing digestive enzyme activity, and secondly, by physically interacting with starch molecules to form a barrier that blocks enzyme access [26,48,49]. Furthermore, the addition of insect powder reduced the gas retention capacity of the gluten network during fermentation and steaming, resulting in a denser crumb structure. This compact structure, which is more resistant to breakdown during digestion, consequently lowered starch digestibility [50]. Zhao et al. [51] reported similar changes when producing CSB with kiwi starch. They attributed these changes to the dense microstructure formed after kiwi starch substitution, which impeded physical contact between starch and digestive enzymes, thereby causing a decreased starch hydrolysis rate of CSB.

### 3.6. FTIR Spectroscopy

**Figure 2:**
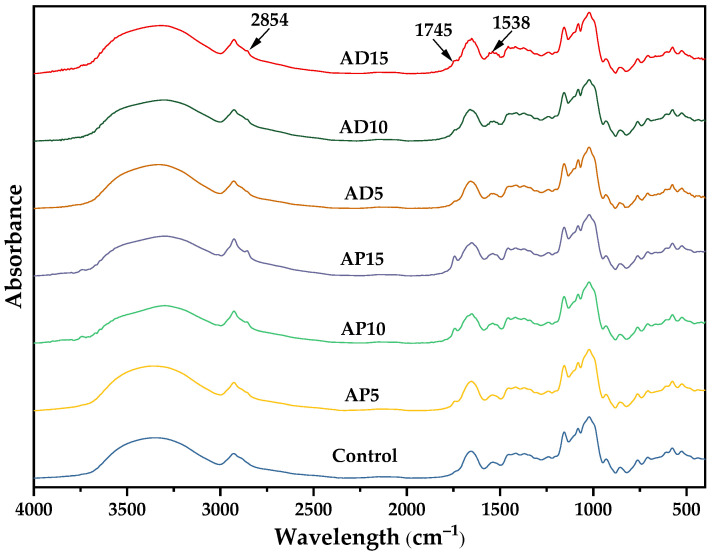
Fourier transform infrared spectra of CSB fortified with *Antheraea pernyi* or *Acheta domesticus* powder. Control represents the CSB without insect powder fortification. AP5, AP10, and AP15 represent the CSB fortified with 5%, 10%, and 15% of *Antheraea pernyi* pupae powder, respectively; AD5, AD10, and AD15 represent the CSB fortified with 5%, 10%, and 15% of *Acheta domesticus* powder, respectively.

Figure 2 shows the FTIR spectra of CSB fortified with varying levels of insect powder. A broad and pronounced infrared absorption peak was observed in the 3700–3000 cm^−1^ range across all CSB samples, characteristic of the O–H stretching vibrations inherent to starch [52]. The peak at 1538 cm^−1^, associated with the amide II band of proteins also appeared in all CSB samples, suggesting molecular interactions between starch and proteins during CSB preparation [53]. A notable difference in the FTIR spectra was the exclusive presence of peaks at 2854 cm^−1^ (C–H stretching vibration) and 1745 cm^−1^ (C=O stretching vibration) [54] in AP- and AD-fortified CSB. These characteristic peaks, which were more pronounced at higher AP addition levels, are indicative of lipids and confirm the successful incorporation of insect-derived lipids into the CSB matrix. Furthermore, their presence suggests that these lipids may have participated in forming starch–lipid complexes or other intermolecular associations during thermal processing.

### 3.7. E-Nose Analysis

**Figure 3:**
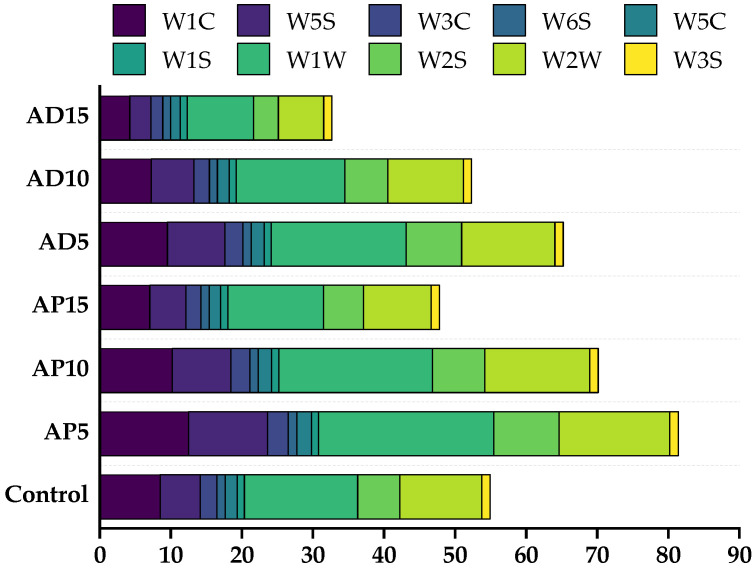
The E-nose sensor response signal values for CSB. Control represents the CSB without insect powder fortification. AP5, AP10, and AP15 represent the CSB fortified with 5%, 10%, and 15% of *Antheraea pernyi* pupae powder, respectively; AD5, AD10, and AD15 represent the CSB fortified with 5%, 10%, and 15% of *Acheta domesticus* powder, respectively.

To systematically characterize the overall flavor profile of CSB and insect-fortified CSB, an E-nose was employed to detect their volatile components. Figure 3 illustrates the aromatic differences between the control and insect-fortified CSB at various levels. With increasing insect powder addition, the response values of most sensors (excluding W1S and W3S) increased initially and then decreased, suggesting that the lower substitution levels (5%) of insect powder introduced higher amounts of aromatic components, nitrogen oxides, hydrides, alcohols, aldehydes, ketones, and sulfides into CSB, whereas a higher substitution level (10–15%) may have led to their partial degradation or masking by other flavor-active constituents. To have a more accurate response to the dissimilarity in odorant makeup among the CSB with varying AP or AD additions, further exploration in combination with GC-MS is needed.

### 3.8. Volatile Flavor Compounds

Flavor is a critical determinant of consumer acceptance and overall quality evaluation of CSB [55]. Therefore, this study used GC-MS for the analysis of the volatile flavor compounds in CSB with varying levels of edible insect powder. Fifty-seven VOCs were detected in the samples, comprising aldehydes, alcohols, hydrocarbons, ketones, esters, benzenes, pyrazines, terpenes, and furans. As shown in Table 7, the incorporation of the two edible insects induced a greater diversity and complexity of VOCs compared to the control CSB. Seven new VOCs were identified in the AP-fortified CSB samples, including (E,Z)-2,6-nonadienal, heptanal, octanal, 2,6-dimethyl-undecane, (E,E)-3,5-Octadien-2-one, acetophenone, and 2-methoxy-4-vinylphenol, whereas in the AD-fortified samples, thirteen new VOCs were detected, including (E)-2-heptenal, 2-butyl-2-octenal, heptanal, hexanal, 1-undecanol, 2,3-nonanedione, 2-decanone, (E)-3-octen-2-one, 2,3-dimethyl-5-ethylpyrazine, 2,5-dimethyl-pyrazine, 2,5-dimethyl-3-(3-methylbutyl)-pyrazine, 3,5-diethyl-2-methyl-pyrazine, and 3-ethyl-2,5-dimethyl-pyrazine. These new VOCs, primarily aldehydes, ketones, and pyrazines, were likely generated by promoted Maillard reaction and lipid oxidation, thereby enriching the overall flavor profile of CSB.

**Figure 4:**
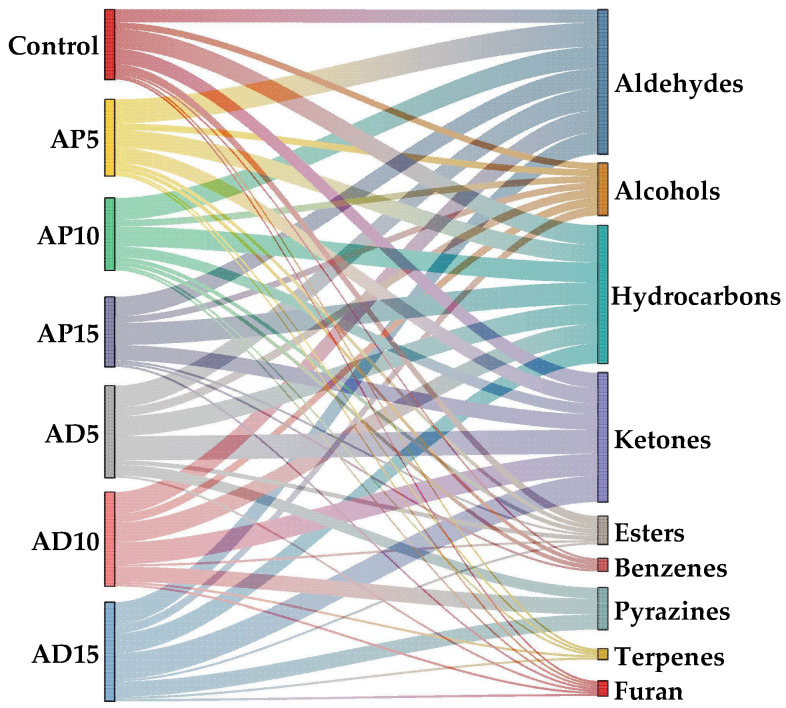
Sankey diagram of 57 volatile flavor compound species.

As illustrated in Figure 4, aldehydes were the dominant volatile compounds in both abundance and concentration across all sample groups, establishing them as the principal contributors to the CSB’s aroma. Aldehydes are predominantly generated through the oxidation of unsaturated fatty acids. Specifically, hexanal (grass) [56] and (E)-2-octenal (fatty, nutty, and roasted) [57] are primarily derived from linoleic acid oxidation [58]. Nonanal (rose and citrus) [59], heptanal (fatty, greasy, and fruity) [60] and octanal (fruity and fatty) [60] are secondary oxidation products of oleic acid, contributing pleasant fruity and fatty aromas [60,61]. The AP-fortified CSB contained high levels of nonanal, decanal (citric) [62], (E,Z)-2,6-nonadienal (fatty and green) [63], heptanal, and octanal, enhancing fruity flavor. Conversely, the AD-fortified CSB had richer concentrations of benzeneacetaldehyde (hawthorne and honey) [64,65], (E)-2-octenal, (E)-2-heptenal, 2-butyl-2-octenal (sweetish, metallic, and citrus) [66], and hexanal. The elevated nonanal content in AP-fortified CSB may be associated with the high oleic acid concentration in AP [67]. Ketones are primarily produced from lipid oxidation and Maillard reaction [60]. The elevated relative content in AD-fortified CSB may be due to the greater amount of protein in AD, which promoted Maillard-derived ketone formation. Specifically, 2-decanone, 2-nonanone (rose and tea) [68], 2-octanone, and (E)-3-octen-2-one were present at higher levels in AD-fortified CSB, likely resulting from the oxidation of free fatty acids [61]. Hydrocarbons, formed through the cleavage of fatty acid alkoxy radicals [60]. However, as a result of their generally high odor thresholds, hydrocarbons contributed little to the direct flavor of CSB but may still act as a complement to modify the overall flavor background and perception [69].

The overall concentration of alcohols decreased after adding insect powder, a trend consistent with their oxidation into corresponding aldehydes, thereby explaining simultaneous aldehyde increase [70]. Among key alcohols detected across all samples, ethanol was more abundant in the control than in the AP- and AD-fortified CSB, whereas 1-octen-3-ol (mushroom) [60] was detected at higher levels in the AD- fortified CSB. Additionally, the ester level in the control was higher than that in the AP- and AD-fortified CSB [71]. Esters play a critical role in flavor modulation attributed to their low odor thresholds. Esters derived from short-chain acids mainly impart fruity notes, whereas those formed from long-chain acids contribute mild fatty undertones [72]. Meanwhile, The Maillard reaction products, such as pyrazines [73], 2-pentylfuran (green bean and floral odor) [32,56], were more abundant in AD-fortified CSB compared to both the control and AP-fortified samples.

### 3.9. E-Tongue Analysis

**Figure 5:**
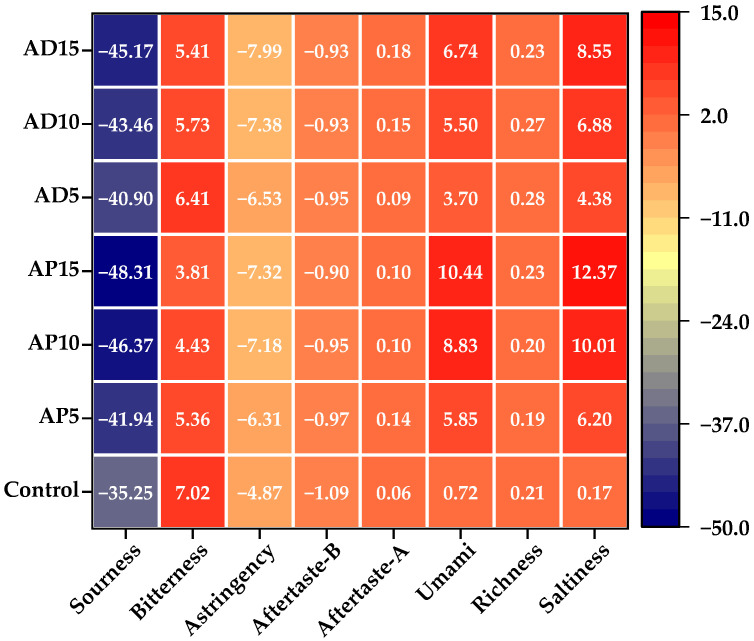
Heat map of the E-tongue for CSB. Control represents the CSB without insect powder fortification. AP5, AP10, and AP15 represent the CSB fortified with 5%, 10%, and 15% of *Antheraea pernyi* pupae powder, respectively; AD5, AD10, and AD15 represent the CSB fortified with 5%, 10%, and 15% of *Acheta domesticus* powder, respectively.

The E-tongue has been widely applied in food analysis owing to its extremely high sensitivity [74]. E-tongue sensor responses toward flavor compounds in CSB with differential insect powder incorporation was visualized in the heat map presented in Figure 5. In this representation, deeper red shades indicate higher response values, whereas deeper blue hues correspond to lower values. Among the eight taste attributes evaluated, the values for sourness, astringency, and aftertaste-B in all samples were below the detection threshold of the reference solution. Therefore, these tastes were considered negligible in the CSB samples. Notably, the incorporation of either AP or AD enhanced the umami and saltiness of CSB, which is likely resulting from the abundance of umami amino acids (e.g., glutamate) and minerals (e.g., sodium and potassium) in the insect powder [14,75].

**Figure 6:**
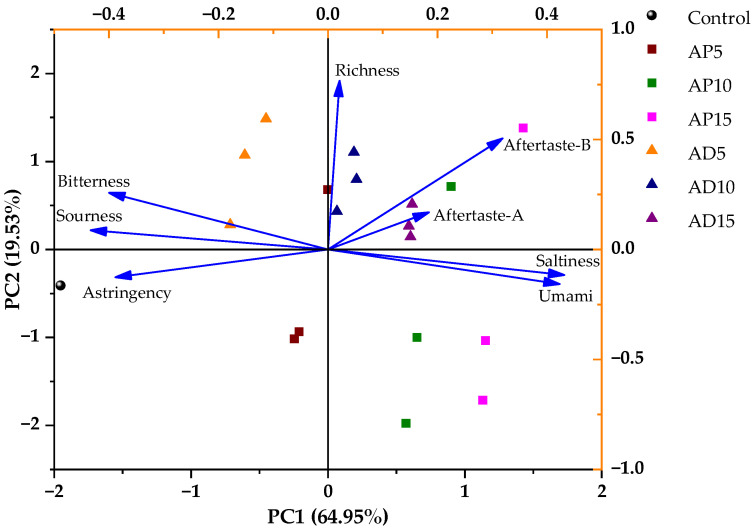
Principal component analysis score and loading plot of the E-tongue for CSB. Control represents the CSB without insect powder fortification. AP5, AP10, and AP15 represent the CSB fortified with 5%, 10%, and 15% of *Antheraea pernyi* pupae powder, respectively; AD5, AD10, and AD15 represent the CSB fortified with 5%, 10%, and 15% of *Acheta domesticus* powder, respectively.

To further explore the differences in taste characteristics of CSB with varying insect powder content, PCA was applied to the E-tongue response values of each sample group. As illustrated in Figure 6, PC1 and PC2 explained 64.95% and 19.53% of the total variance, respectively, cumulatively explaining 84.48% of the variance—sufficient to capture most of the sample information [60]. PC1 was positively correlated with richness, aftertaste-A, aftertaste-B, saltiness, and umami, while it showed negative correlations with bitterness, sourness, and astringency. Among them, sourness and saltiness contributed more strongly to PC1, while richness was more influential on PC2. The shorter arrow length of aftertaste-A suggested its relatively lower contribution to the overall taste profile of CSB. The distribution of sample points correlated with distinct taste profiles. For example, AD15 displayed more pronounced aftertaste-A, while AP15 exhibited stronger umami and saltiness. It is worth noting that E-tongue analysis was used not for directly determining sensory acceptability, but for detecting subtle differences in taste characteristics among the CSB samples.

## 4. Conclusions

*A. pernyi* pupae powder (AP) and *A. domesticus* powder (AD) are rich sources of antioxidants and protein, displayed huge potential as functional ingredients in Chinese steamed bread (CSB). Adding AP and AD to wheat flour resulted in CSB with higher protein, fat, and ash contents compared to the control. Texture characterization revealed that the insect powder supplementation led to increased hardness and chewiness, which may be due to the increased protein content interfering with gluten network formation. In addition, increasing the level of AP or AD fortification enhanced the antioxidant activity and reduced the starch digestibility, which may a consequence of the presence of protein and polyphenols in the insect powder. The partial replacement of wheat flour by AP or AD also enriched the volatile flavor profile of CSB, enhanced umami and saltiness perception, and reduced bitterness. This study demonstrated that AP and AD are sustainable and effective ingredients for producing CSB with improved nutritional value, bioactive content, flavor profile, and estimated glycemic index, thereby offering consumers greater diversity in CSB choices. Overall, this study provided a theoretical foundation and practical framework for developing insect powder-fortified wheat-based products, supporting the application of edible insects in nutritional and functional enhancement.

While insect powder enhances nutrition, its high concentration compromises the sensory experience by increasing hardness and altering flavor—a critical impediment for consumer products. Future work must therefore pivot towards sensory science. A dedicated sensory evaluation is therefore recommended in future studies to determine consumer acceptance of these novel products, which is a critical step towards commercialization. Meanwhile, research should branch into technical optimization of formulations and processing and alignment of these optimized recipes with regional regulatory frameworks to ensure market viability.

## Figures and Tables

**Table 1:** Ten sensors and their corresponding sensitive substances.

Sensor Name	Aroma Type
W1C	Aromatic components
W5S	Nitrogen oxides
W3C	Aromatic and ammonia components
W6S	Hydrides
W5C	Aromatic components (short-chain alkanes)
W1S	Methyl compounds
W1W	Inorganic sulfides, terpenes
W2S	Alcohols, aldehydes, and ketones
W2W	Aromatic components (organic sulfides)
W3S	Long-chain alkanes

**Table 2:** Nutritional composition of CSB fortified with *Antheraea pernyi* pupae or *Acheta domesticus* powder.

Sample	Moisture (%) *	Protein (%) **	Fat (%) **	Ash (%) **	Carbohydrate (%) **
Control	38.92 ± 0.28 a	11.87 ± 0.35 d	0.51 ± 0.04 e	0.40 ± 0.06 d	87.23 ± 0.36 a
AP5	38.41 ± 0.45 a	15.26 ± 1.87 c	0.88 ± 0.08 cd	0.65 ± 0.10 cd	83.21 ± 1.74 b
AP10	35.11 ± 0.29 d	17.69 ± 3.13 bc	1.84 ± 0.27 b	0.82 ± 0.07 bc	79.65 ± 3.12 c
AP15	34.62 ± 0.55 d	19.17 ± 0.10 ab	2.48 ± 0.34 a	1.07 ± 0.22 b	77.29 ± 0.16 cd
AD5	37.49 ± 1.72 ab	15.18 ± 0.45 c	0.67 ± 0.02 de	0.82 ± 0.31 bc	83.32 ± 0.30 b
AD10	36.74 ± 0.75 bc	18.68 ± 0.16 ab	1.04 ± 0.04 c	0.96 ± 0.24 bc	79.32 ± 0.35 c
AD15	35.88 ± 0.37 cd	20.91 ± 0.67 a	1.71 ± 0.04 b	1.65 ± 0.28 a	75.72 ± 0.83 d

* percent of wet weight; ** percent of dry weight. Values are expressed as mean ± standard deviation (*n* = 3), and values with different lowercases in the same column are considered significant difference (*p* < 0.05). Control represents the CSB without insect powder fortification. AP5, AP10, and AP15 represent the CSB fortified with 5%, 10%, and 15% of *Antheraea pernyi* pupae powder, respectively; AD5, AD10, and AD15 represent the CSB fortified with 5%, 10%, and 15% of *Acheta domesticus* powder, respectively.

**Table 3:** Color of fresh CSB fortified with *Antheraea pernyi* pupae or *Acheta domesticus* powder.

Sample	L*	a*	b*
Control	77.04 ± 3.40 a	0.54 ± 0.80 d	17.27 ± 0.72 c
AP5	73.66 ± 4.72 ab	1.57 ± 2.10 cd	21.55 ± 1.13 bc
AP10	69.97 ± 2.16 bc	2.13 ± 1.04 bcd	26.21 ± 0.50 ab
AP15	66.27 ± 2.76 c	3.56 ± 2.41 abc	29.68 ± 7.73 a
AD5	66.04 ± 0.50 c	2.73 ± 1.34 bcd	19.39 ± 0.44 bc
AD10	60.08 ± 1.41 d	4.89 ± 1.13 ab	22.17 ± 3.68 bc
AD15	56.67 ± 0.96 d	6.29 ± 1.21 a	22.47 ± 3.56 bc

Values are expressed as mean ± standard deviation (*n* = 3), and values with different lowercases in the same column are considered significant difference (*p* < 0.05). Control represents the CSB without insect powder fortification. AP5, AP10, and AP15 represent the CSB fortified with 5%, 10%, and 15% of *Antheraea pernyi* pupae powder, respectively; AD5, AD10, and AD15 represent the CSB fortified with 5%, 10%, and 15% of *Acheta domesticus* powder, respectively.

**Table 4:** Textural properties of CSB fortified with *Antheraea pernyi* pupae or *Acheta domesticus* powder.

Sample	Hardness (g)	Springiness	Cohesiveness	Chewiness	Resilience
Control	11,237.10 ± 1871.79 c	0.85 ± 0.04 a	0.75 ± 0.01 a	4628.77 ± 268.05 c	0.38 ± 0.04 a
AP5	13,913.11 ± 1146.17 bc	0.71 ± 0.09 ab	0.64 ± 0.06 a	5588.15 ± 425.44 c	0.35 ± 0.03 ab
AP10	14,412.83 ± 2391.77 bc	0.70 ± 0.03 ab	0.64 ± 0.05 a	6576.18 ± 426.91 bc	0.33 ± 0.03 ab
AP15	19,626.01 ± 4124.43 ab	0.64 ± 0.09 b	0.50 ± 0.08 b	7093.01 ± 295.32 bc	0.31 ± 0.02 bc
AD5	16,274.13 ± 904.81 bc	0.84 ± 0.02 a	0.71 ± 0.02 a	5717.18 ± 546.62 c	0.27 ± 0.03 cd
AD10	18,174.92 ± 1643.80 b	0.83 ± 0.01 a	0.71 ± 0.02 a	9666.90 ± 1155.80 b	0.23 ± 0.02 de
AD15	25,111.6 ± 7 021.99 a	0.74 ± 0.15 ab	0.66 ± 0.14 a	15,846.65 ± 5116.60 a	0.20 ± 0.05 e

Values are expressed as mean ± standard deviation (*n* = 5), and values with different lowercases in the same column are considered significant difference (*p* < 0.05). Control represents the CSB without insect powder fortification. AP5, AP10, and AP15 represent the CSB fortified with 5%, 10%, and 15% of *Antheraea pernyi* pupae powder, respectively; AD5, AD10, and AD15 represent the CSB fortified with 5%, 10%, and 15% of *Acheta domesticus* powder, respectively.

**Table 5:** Antioxidant activities of CSB fortified with *Antheraea pernyi* pupae or *Acheta domesticus* powder.

Sample	TPC (mg GAE/100 g)	TFC (mg RE/100 g)	DPPH (%)	ABTS (%)
Control	5.63 ± 0.10 f	0.45 ± 0.26 f	13.38 ± 0.56 f	34.98 ± 1.52 f
AP5	16.58 ± 0.36 c	4.12 ± 0.52 e	46.41 ± 2.76 c	89.37 ± 2.73 b
AP10	27.30 ± 1.46 b	6.82 ± 0.40 d	58.97 ± 1.84 b	97.99 ± 0.41 a
AP15	32.66 ± 1.01 a	9.97 ± 0.15 b	65.55 ± 1.88 a	98.56 ± 0.24 a
AD5	7.85 ± 0.26 e	3.85 ± 0.52 e	29.60 ± 2.72 e	45.30 ± 1.53 e
AD10	12.04 ± 0.55 d	8.48 ± 0.55 c	40.06 ± 3.81 d	59.44 ± 1.32 d
AD15	15.64 ± 0.33 c	12.59 ± 1.29 a	42.15 ± 3.52 cd	77.50 ± 2.00 c

Values are expressed as mean ± standard deviation (*n* = 3), and values with different lowercases in the same column are considered significant difference (*p* < 0.05). Control represents the CSB without insect powder fortification. AP5, AP10, and AP15 represent the CSB fortified with 5%, 10%, and 15% of *Antheraea pernyi* pupae powder, respectively; AD5, AD10, and AD15 represent the CSB fortified with 5%, 10%, and 15% of *Acheta domesticus* powder, respectively. TPC: total phenolic content; TFC: total flavonoid content.

**Table 6:** In vitro starch digestibility of CSB fortified with *Antheraea pernyi* pupae or *Acheta domesticus* powder.

Sample	RDS (%)	SDS (%)	RS (%)	eGI
Control	58.87 ± 1.71 a	32.11 ±0.37 d	9.03 ± 1.85 e	88.90 ± 0.31 a
AP5	55.43 ± 0.35 b	33.86 ± 0.46 bc	10.71 ± 0.81 d	85.20 ± 0.72 b
AP10	50.97 ± 0.27 c	34.69 ± 0.55 b	14.34 ± 0.49 c	76.48 ± 0.36 d
AP15	45.48 ± 1.47 d	36.16 ± 1.44 a	18.36 ± 0.16 b	70.63 ± 0.26 f
AD5	54.68 ± 0.46 b	33.37 ± 0.61 c	11.95 ± 0.22 d	83.68 ± 1.59 c
AD10	50.71 ± 0.19 c	34.52 ± 0.26 bc	14.77 ± 0.15 c	74.48 ± 0.26 e
AD15	43.43 ± 0.19 e	36.44 ± 0.32 a	20.13 ± 0.14 a	67.82 ± 0.05 g

Values are expressed as mean ± standard deviation (*n* = 3), and values with different lowercases in the same column are considered significant difference (*p* < 0.05). Control represents the CSB without insect powder fortification. AP5, AP10, and AP15 represent the CSB fortified with 5%, 10%, and 15% of *Antheraea pernyi* pupae powder, respectively; AD5, AD10, and AD15 represent the CSB fortified with 5%, 10%, and 15% of *Acheta domesticus* powder, respectively. RDS: rapidly digestible starch; SDS: slowly digestible starch; RS: resistant starch; eGI: estimated glycemic index.

**Table 7:** Volatile flavor compounds of CSB fortified with *Antheraea pernyi* pupae or *Acheta domesticus* powder.

	Control	AP5	AP10	AP15	AD5	AD10	AD15
**Aldehydes**	**36.59**	**71.74**	**69.67**	**71.91**	**41.97**	**26.92**	**36.46**
Nonanal	16.57	51.55	54.63	57.00	19.56	11.80	19.03
2-Nonenal, (E)-	10.12	7.18	4.13	3.30	5.38	1.97	2.27
Benzaldehyde	3.45	0.46	0.57	0.57	2.62	2.34	3.61
Decanal	3.23	5.75	4.15	5.21	4.86	3.56	5.05
2-Octenal, (E)-	1.80	0.46	0.24	0.05	1.90	0.96	1.07
Benzeneacetaldehyde	1.42	1.45	1.97	1.51	2.01	2.23	1.84
Heptanal	-	0.73	1.11	1.05	0.30	0.23	0.80
2-Heptenal, (E)-	-	0.17	-	-	2.28	0.51	1.07
Hexanal	-	0.50	0.58	-	2.46	2.70	0.93
2,6-Nonadienal, (E,Z)-	-	2.53	1.61	1.85	-	-	-
Octanal	-	0.96	0.68	1.37	-	-	-
2-Octenal, 2-butyl-	-	-	-	-	0.60	0.62	0.79
**Alcohols**	**10.25**	**3.02**	**3.51**	**1.98**	**6.28**	**8.74**	**4.95**
Ethanol	4.92	0.19	1.60	-	1.32	1.99	0.84
1-Octen-3-ol	3.17	2.21	1.36	1.34	2.89	5.46	3.66
1-Hexanol	2.16	0.62	-	0.16	1.66	1.03	0.16
1-Undecanol	-	-	-	-	0.41	0.26	0.29
1-Hexanol, 2-ethyl-	-	-	0.55	0.48	-	-	-
**Hydrocarbons**	**25.14**	**17.26**	**20.23**	**20.63**	**22.46**	**26.88**	**24.39**
Dodecane	4.17	2.57	5.18	5.55	4.22	4.77	5.66
Dodecane, 2,6,10-trimethyl-	0.24	0.06	1.10	0.91	0.68	0.25	0.28
Dodecane, 4,6-dimethyl-	1.93	-	-	-	0.06	1.12	1.04
Heptadecane	2.55	-	-	0.93	0.73	-	-
Hexadecane	4.10	3.96	2.19	2.00	3.98	3.86	2.07
Nonadecane	-	-	0.84	0.25	-	-	-
Tetradecane	6.85	6.98	6.32	6.08	8.91	10.23	10.21
Tridecane	3.10	2.11	2.52	2.56	2.10	2.83	2.39
Tridecane, 3-methyl-	1.23	1.21	1.48	1.71	1.59	1.49	1.39
Undecane, 2,6-dimethyl-	-	0.26	0.17	0.20	-	0.32	0.31
Undecane, 3-methyl-	0.97	0.11	0.43	0.44	0.19	2.01	1.04
**Ketones**	**9.22**	**3.41**	**1.78**	**1.97**	**13.26**	**14.54**	**15.59**
2(3H)-Furanone, dihydro-5-pentyl-	1.20	0.46	-	-	0.26	-	0.37
2,3-Nonanedione	-	-	-	-	0.13	0.21	0.29
2-Decanone	-	-	-	0.29	2.49	3.67	4.06
2-Heptanone	-	-	-	-	-	1.48	2.79
2-Nonanone	1.08	-	-	0.38	2.74	3.49	3.00
2-Octanone	1.29	-	-	-	2.36	1.70	0.8
3,5-Octadien-2-one	0.67	0.45	0.89	0.60	0.99	0.86	0.18
3,5-Octadien-2-one, (E,E)-	-	0.75	0.58	0.29	-	-	-
3-Nonen-2-one	-	-	-	-	0.25	0.07	0.38
3-Octen-2-one	1.25	0.24	0.08	0.20	-	-	-
3-Octen-2-one, (E)-	-	-	-	-	2.31	2.14	2.28
5,9-Undecadien-2-one, 6,10-dimethyl-, (Z)-	2.95	1.22	-	-	1.26	0.71	0.81
5-Hepten-2-one, 6-methyl-	0.78	0.13	0.06	0.19	0.12	0.21	0.24
Acetophenone	-	0.16	0.17	0.02	0.35	-	0.39
**Esters**	**7.78**	**0.94**	**0.64**	**0.65**	**0.69**	**0.29**	**0.l0**
Acetic acid, 2-phenylethyl ester	1.98	-	-	-	-	-	-
Acetic acid, hexyl ester	1.19	-	-	-	-	-	-
Octanoic acid, ethyl ester	0.66	0.29	0.20	-	0.30	-	-
Pentadecanoic acid, 14-methyl-, methyl ester	3.95	0.65	0.44	0.65	0.39	0.29	0.10
**Benzenes**	**0.34**	**0.68**	**1.16**	**0.71**	**-**	**-**	**-**
2-Methoxy-4-vinylphenol	-	0.43	0.69	0.71	-	-	-
Styrene	0.34	0.25	0.47	-	-	-	-
**Pyrazines**	**-**	**-**	**-**	**-**	**5.05**	**9.68**	**13.10**
Pyrazine, 2,3-dimethyl-5-ethyl-	-	-	-	-	0.16	0.33	0.25
Pyrazine, 2,3-diethyl-5-methyl-	-	-	-	-	-	0.26	0.25
Pyrazine, 2,5-dimethyl-	-	-	-	-	0.28	0.49	1.63
Pyrazine, 2,5-dimethyl-3-(3-methylbutyl)-	-	-	-	-	1.23	1.44	1.83
Pyrazine, 3,5-diethyl-2-methyl-	-	-	-	-	0.16	0.39	0.50
Pyrazine, 3-ethyl-2,5-dimethyl-	-	-	-	-	3.22	6.52	8.15
Pyrazine, trimethyl-	-	-	-	-	-	0.25	0.49
**Terpene**	**0.97**	**0.16**	**0.29**	**-**	**-**	**1.44**	**0.61**
D-Limonene	0.97	0.16	0.29	-	-	1.44	0.61
**Furan**	**9.70**	**2.79**	**2.72**	**2.16**	**10.31**	**11.51**	**4.79**
Furan, 2-pentyl-	9.70	2.79	2.72	2.16	10.31	11.51	4.79

Control represents the CSB without insect powder fortification. AP5, AP10, and AP15 represent the CSB fortified with 5%, 10%, and 15% of *Antheraea pernyi* pupae powder, respectively; AD5, AD10, and AD15 represent the CSB fortified with 5%, 10%, and 15% of *Acheta domesticus* powder, respectively.

## Data Availability

The original contributions presented in the study are included in the article/Supplementary Materials, further inquiries can be directed to the corresponding authors.

## Supplementary Materials

The following supporting information can be downloaded at: https://www.mdpi.com/article/10.3390/foods14223956/s1, Table S1. Proximate composition of raw materials [12,34].
