# Supplementary material for: Nutritional and Functional Enhancement of Chinese Steamed Bread Through Incorporation of Acheta domesticus and Antheraea pernyi Pupae Powders
Source: Foods. 2025 Nov 19;14(22):3956. doi: 10.3390/foods14223956 (PMC12652232; doi:10.3390/foods14223956)
Supplement: Supplementary file 1 [file foods-14-03956-s001.zip › foods-3905818-supplementary.pdf]

Table S1. Proximate composition of raw materials [12,34].

Sample	Moisture (%)	Protein (%)	Fat (%)	Ash (%)	Fiber (%)	Carbohydrate (%)
Antheraea pernyi pupae powder	-	57.31 ± 0.65 [b]	28.18 ± 0.63 [a]	1.98 ± 0.08 [b]	-	12.54 ± 0.26 [a]
Acheta domesticus powder	2.35 ± 0.01	60.40 ± 0.13 [a]	20.68 ± 0.15 [b]	2.24 ± 0.19 [a]	5.89 ± 0.33	7.99 ± 0.11 [b]

Values are expressed as mean ± standard (n = 3). Different letters within the same column represent significant differences between samples ($p < 0.05$).
